# Nutrient profiling and degree of food processing of child-targeted packaged foods in Türkiye: An urgent call for policy action

**DOI:** 10.1371/journal.pone.0330687

**Published:** 2025-08-20

**Authors:** Lütfiye Parlak-Yetişen, Derya Dikmen

**Affiliations:** Department of Nutrition and Dietetics, Faculty of Health Sciences, Hacettepe University, Ankara, Türkiye; Necmettin Erbakan Üniversitesi: Necmettin Erbakan Universitesi, TÜRKIYE

## Abstract

This study aimed to evaluate the nutritional quality and degree of processing of packaged foods and beverages marketed to children in Türkiye using nutrient profiling models and the NOVA classification. A cross-sectional study was conducted by collecting packaging and label data from child-targeted food and beverage products available in grocery stores.The study was carried out in 23 grocery stores in Ankara, Türkiye. A total of 775 packaged food and beverage products marketed to children aged ≥3 years were analyzed. The products were assessed for compliance with four different nutrient profile models (NPMs) and classified according to the NOVA food processing system. Findings indicate that 93.2% of products did not comply with the WHO NPM-2023 criteria and should not be marketed to children. Additionally, the majority of these products were classified as Nutri-Score groups D and E (70%) and ultra-processed (92.7%). Ultra-processed foods had a significantly lower nutritional quality (p < 0.001) according to the Nutri-Score. A significant difference was observed between NOVA classification and product categorization under the NPMs (p < 0.001). Most packaged foods and beverages marketed to children in Türkiye are ultra-processed and nutritionally inadequate. According to WHO and other NPMs, these products should not be marketed to or consumed by children. There is an urgent need for policy interventions to restrict the marketing of unhealthy packaged foods targeted at children.

## Introduction

The prevalence of childhood obesity has reached alarming levels worldwide in recent years, with numbers continuing to rise as the market for children-targeted food products expands [1,2]. The World Health Organization (WHO) indicated that 35 million children under the age of five were overweight in 2022 [3]. In the WHO Global Nutrition Goals-2025, it is estimated that if the current trends persist, the global prevalence of overweight and obese children under the age of five will rise from 7% in 2012 to 11% by 2025 [4]. In Türkiye, the prevalence of obesity, including overweight, in children was reported as 22.4% [5]. Children’s exposure to unhealthy food and beverage marketing is a significant risk factor for childhood obesity and non-communicable diseases [6]. There is substantial evidence that most foods marketed to children are unhealthy, ultra-processed, and high in sodium, sugar, and fat, with low nutritional quality [7,8]. The consumption of ultra-processed foods among children has risen, largely due to the influence of food marketing [9,10]. Studies indicate that excessive consumption of ultra-processed foods in children is linked to food addiction, obesity, impaired adiposity parameters, disrupted lipid profiles, malnutrition, increased waist circumference, and dental caries [11]. A key driver of this overconsumption is the aggressive marketing strategies employed by food companies to promote these products [12]. Various persuasive techniques are used to influence children’s food attitudes, preferences, and consumption behaviors. These products are marketed across multiple platforms, including television commercials, digital media, physical store environments, and product packaging [12,13]. Food packaging, in particular, incorporates a wide range of marketing tactics, such as licensed characters, mascots, cartoon figures, playful themes, unusual flavors and product names, bright colors, toy giveaways, online games, health and nutrition claims, distinctive product shapes, and the use of the word “children” in brand names [13].

Available evidence suggests marketing influences children’s beliefs, attitudes, behaviors, dietary habits, and overall health and well-being [7]. Due to their cognitive development, children are particularly vulnerable to persuasive messages used in foods marketing. Consequently, the marketing of unhealthy foods to children remains a significant public health concern despite recommendations from the WHO and other international organizations to halt the rise in obesity and reduce the marketing of unhealthy, highly processed foods and beverages to children [14–18].

However, although packaging is a major source of children’s exposure to unhealthy food marketing, it is an area often neglected by national and international regulations that focus on advertising and represents a dangerous gap that can be exploited by the food industry [19]. Therefore, there is a need to investigate the nutrient profile and processing characteristics of packaged industrial foods marketed to children from a child health perspective.

Nutrient profiling is the science of classifying or ranking foods based on their nutritional content [20]. Many different models have been developed, each using different nutrients and different thresholds and classifications [21–24]. Nutrient Profiling Models (NPMs) are used for variety purposes, both regulatory and non regulatory including consumer education, developing regulations for health and nutrition claims, guiding food choices in school canteens [20,25]. Additionally nutrient profiling is recognized as an objective tool for implementing restrictions on food marketing to children [20,25].

To the best of our knowledge, no previous studies have evaluated the nutritional quality and degree of processing of packaged foods and beverages marketed specifically to children aged ≥3 years in Türkiye. Therefore, the present study addresses the following research question: “What is the nutritional quality and degree of processing of packaged foods and beverages targeted at children aged ≥3 years, based on internationally recognized nutrient profile models?” To answer this question, we assessed these products using four nutrient profile models designed to restrict marketing of unhealthy foods to children: the Turkish Nutrient Profile Model (TR NPM) [26], the WHO Nutrient Profile Model-2023 (WHO NPM-2023) [21], the UK FSA/Ofcom Nutrient Profile Model (UK FSA/Ofcom NPM) [22], and the Nutri-Score [27,28], along with evaluating their degree of processing using the NOVA classification [29,30].

## Methods

### Data collection

This cross-sectional study involved visits to 23 grocery stores in Ankara, including supermarket chains with nationwide branches. A total of 781 packaged food and beverage products marketed to children aged ≥3 years were identified and analyzed. The front and back packaging, labeling information of food and beverages were systematically documented through photographs and recorded for analysis to excel spreadsheet. To avoid duplication, different package sizes of the same product were not recorded separately. The recorded products were then classified into 18 food groups based on the categorization outlined in the WHO European-2023 framework [21].

Products in the grocery store were considered to be targeted at children and included in the sample if at least one of the following conditions was met [31];

- The product or brand name contains the words “child (ren)”, “kid(s)” or is marketed as being specifically designed for children, if the words “fun”, “game”, “play” are present on the packaging (Pınar Kido, Züber Kidz, Kinder Pingui, Osmo Fun, etc.).
- The packaging featured graphics, drawings, mascots suitable for children; (such as cartoon characters, brand mascots, superheroes)
- The product was associated with children’s television programs or movies, (character license, e.g., Barbie, Disney, Frozen);
- The product had childish names, funny names or fonts aimed at children
- The packaging included interactive activities (puzzles, games) or encourages children to play an online game that challenges their cognitive skills (free game download, code);
- The product offered gifts for children (physical or virtual gifts)
- The packaging contained illustrations or text referencing to children or their attributes (backpack, skateboard, hopscotch, etc.), such as “the perfect snack for kids”.
- The product had unusual or child-targeted shapes, unusual colors, or funny product names, or unusual flavors.

The following food items were excluded from the analysis: infant and toddler formula, follow-on milk, fresh fruit or vegetables, protein powders, dietary supplements, and products that do not require nutrition labeling (i.e., bakery items prepared in-store and store-cut meats).

The products with missing nutritional information, data was obtained from the USDA National Nutrition Database [32] and the Turkish National Food Composition Database (TURKOMP) [33].

### Data analysis

#### Evaluation of food processing degrees.

The classification of processed foods and beverages was determined based on the NOVA system, using ingredient information obtained from the nutrition labels of the child-targeted products. The NOVA classification system is the most widely used framework in the scientific literature for categorizing foods based on their degree of processing [34]. Furthermore, the NOVA classification is recognized as a reliable instrument for nutrition and public health research, policies, as evidenced by its inclusion in the reports of the Food and Agriculture Organization of the United Nations and the Pan American Health Organization [35]. In this study,the NOVA classification of foods was determined through a detailed examination of each ingredient listed on the packaging. Based on this evaluation, all products were classified into one of the following categories according to the definitions of Monteiro et al [29,30]: unprocessed or minimally processed (NOVA 1), processed culinary ingredients (NOVA 2), processed (NOVA 3), and ultra-processed (NOVA 4) (Table 1).

**Table 1:** NOVA classification [29,30].

NOVA 1	They are unprocessed foods like fresh fruits and vegetables. None of these foods add salt, sugar, oils or fats, or other food substances to the original food.
NOVA 2	Their use is in the preparation, seasoning and cooking of group 1 foods. Processed culinary ingredients such as oils and fats, sugar and salt. Also extracted honey included this group.
NOVA 3	These are industrial products made by adding salt, sugar or other substance found in group 2 to group 1 foods, using preservation methods such as canning and bottling, and, in the case of breads and cheeses, using non-alcoholic fermentation.
NOVA 4	These foods are produced through multiple industrial processes. Various additives including flavourings, colourings, emulsifiers, sweeteners, and texturising agents are commonly used to enhance taste, appearance, and texture, making the final product more appealing or hyper-palatable.

#### Evaluation of nutrient profile models.

Nutrient Profiling (NP) is defined as the science of classifying or ranking foods according to their nutritional composition for the purposes of disease prevention and health promotion [36]. Furthermore, the WHO has recognized the importance of NP for a multitude of applications, particularly as a pivotal instrument for implementing restrictions on the marketing of foods to children [25]. Currently, NP is employed as part of numerous global nutrition policy initiatives, with the number of distinct NP models notably increasing in recent years [25].

**Evaluation of Compliance with the WHO Regional Office for Europe Nutrient Profile Model-2023.** Compliance of the collected products with guidelines for marketing to children was assessed according to thresholds established in the updated WHO NPM-2023, revised and published in 2023 [21]. This model was specifically developed for government use across Europe, aiming to restrict the marketing of certain foods to children [25]. The current model includes 18 total categories, encompassing 17 types of foods and beverages. A food item is not permitted for advertising to children if it fails to meet even a single nutrient threshold within these categories (total fat, saturated fat, added sugar, non-sugar sweeteners, sodium, and energy) [21]. Since water products could not be evaluated using this model, they were excluded from the analysis. Consequently, a total of 775 products were assessed

**Evaluation of Compliance with the UK FSA/Ofcom Nutrient Profile Model.** This model is among the earliest nutrient profiling models developed with the aim of regulating the marketing of foods to children [20]. The compliance of the collected products with the UK FSA/ Ofcom NPM was assessed using the scoring system outlined by the model [22]. The scoring system evaluates total fat, saturated fat, total sugar, sodium, fruits, vegetables and nuts, fibre, and protein. The cumulative score classifies foods and beverages as either healthy or less healthy [22].

**Evaluation of Compliance with the Turkish Model for Advertisements of Foods and Beverages Whose Overconsumption Is Not Recommended for Children.** The “Model for Advertisements of Foods and Beverages Whose Overconsumption Is Not Recommended for Children” restricts advertisements of products targeted at children according to Law No. 6112 on the Establishment of Radio and Television Enterprises and Their Media, enacted in 2011 in Türkiye [37]*.* Guidelines for using this model are detailed in the “Guide for Use of the Nutrient Profile Model” developed by the WHO Regional Office for Europe and published by the Turkish Ministry of Health in 2015 [26]*.* The model categorizes foods into three groups based on nutrient content per 100 g: red (not permitted), orange (permitted if specific criteria are met), and green (permitted). Advertising products classified in the red category is banned during children’s programs [37]. Water was excluded from evaluation within this NPM, resulting in a total of 775 products assessed.

**Evaluation of Nutri-Score.** Nutri-Score is a front-of-package nutrition label consisting of five colored categories (A: dark green (the healthiest) to E: dark orange (the unhealthiest)) that represent the overall nutritional quality of a product within its food group [38]. On 29 June 2022, revisions were introduced to categories including ‘general foods’, ‘fats’, ‘nuts and seeds’, and ‘red meat and meat products’ [28]. Further adjustments to the ‘beverages’ category were implemented on 1 February 2023 [27]. These updated algorithms were applied in the present study [27,28]. The nutritional quality of the collected products was assessed by analyzing packaging information and applying the appropriate algorithms to calculate scores and determine the corresponding Nutri-Score category [27,28]. Nutri-Score is calculated according to the energy, total sugar, saturated fat and sodium content per 100 g of the product, with a negative score ranging from 0 to 5, and the fruit, vegetable, pulses and nuts, fiber and protein content, with a positive score ranging from 0 to 10. Subsequently, an algorithm integrates these scores into an overall score. The model has different algorithms for beverages, fats and oils, and cheeses [27,28]. The overall score ranges from −15 (healthiest) to +40 (least healthy) [38].

Nutri-Score is the only recommended labelling scheme that fully aligns with WHO-Europe’s criteria and processes for validation studies necessary for selecting and evaluating front-of-package nutrition labelling [39]. Currently, Nutri-Score is implemented in numerous European countries [38]; however, it has not yet been adopted in Türkiye. This study initially evaluated 781 products, but since water was categorized as “A” without a calculable score, a correlation evaluation was performed on 775 products, excluding water.

### Statistical analysis

The IBM SPSS Statistics program was used for the statistical analysis of the data. Products were categorized according to the WHO NPM-2023 product groups; however, due to insufficient numbers of products within these groups, the analysis was conducted on the entire dataset rather than by individual product groups. The normality of the distribution was assessed. The homogeneity of variances was tested using the Levene’s test. Differences between means were evaluated using an independent sample t-test for comparisons involving two groups, and one-way analysis of variance (ANOVA) for comparisons involving more than two groups. Post hoc analyses were performed to identify specific groups contributing to significant differences observed in ANOVA results. Categorical variables were evaluated using the chi-square test, and correlations between continuous variables were analyzed using Pearson’s correlation test. A p-value below 0.05 (p < 0.05) at a 95% confidence interval was considered statistically significant for all tests.

## Results

### Child-targeted marketing strategies

**Fig 1:**
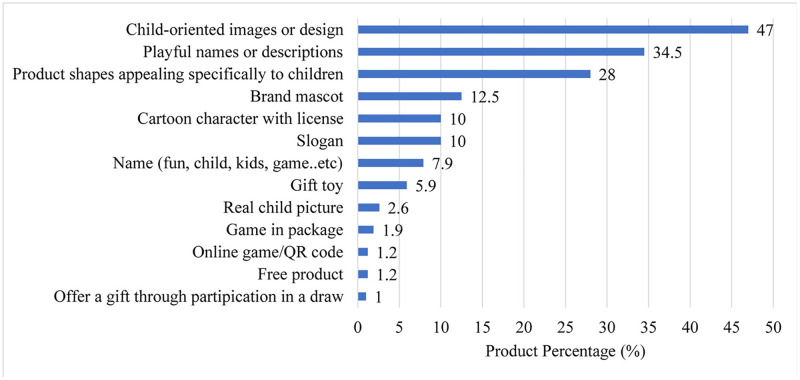
Marketing strategies for children in products.

Packaging and labelling information from 781 products targeted at children was collected through visits to 23 grocery stores. The marketing strategies utilized in these products are presented in the Fig 1. It was observed that 47% of the products contained featured child-oriented images or designs, 34.5% had included playful names or descriptions, and 28% had product shapes appealing specifically to children. The least common marketing strategy was to offer a gift through participation in a draw, identified in only 1% of the products.

### Child-targeted products’ groups

The classification of the 781 child-targeted products collected according to the WHO NPM-2023 Food Categories product categories is presented in the Table 2. The 38% of the packaged products targeted at children on the market consisted of chocolate, confectionery, energy bars and sweets. Cakes and sweet biscuits constituted 23% of the products. Categories such as practical ready-to-eat foods (including bread and bread products), meat, fish, poultry (fresh, frozen and processed), vegetables, fruits, legumes, plant-based foods and meat analogues, and sauces were not marketed as products specifically targeting children.

**Table 2:** Child-targeted products according to WHO NPM-2023 product categories.

Product Categories (WHO)	n	%
1. Chocolate and sugar confectionery, energy bars and sweet toppings and desserts	297	38.0
2. Cakes,sweet biscuits and pastries; other sweet bakery wares; and dry mixes for making such	180	23.0
3. Savoury snacks	65	8.3
4. Beverages	101	12.9
5. Edible ices	30	3.9
6. Breakfast cereals	16	2.0
7. Yogurt, sour milk, cream and similar foods	52	6.8
8. Cheese	15	1.9
9. Ready-made and convenience foods and composite dishes	0	0.0
10. Butter and other fats and oil	1	0.1
11. Bread, bread products and crisp breads	0	0.0
12. Fresh or dried pasta, noodles, rice and grains	7	0.9
13. Fresh and frozen meat, poultry, fish and similar	0	0.0
14. Processed meat, poultry, fish and similar	0	0.0
15. Fresh and frozen fruit, vegetables and legumes	0	0.0
16. Processed fruit, vegetables and legumes	17	2.2
17. Plant-based food/meat analogues	0	0.0
18. Sauces,dips and dressings	0	0.0
**Total**	**781**	**100.0**

### Assessment of food processed degree

Among the child-targeted products, 93.4% were classified as ultra-processed (NOVA 4). A statistically significant difference (p < 0.001) was observed between the mean nutrient values of the products across the different NOVA groups of child-targeted products for which label information was collected. Significant differences between the groups were evaluated by post hoc tests and the results are presented in Table 3.

**Table 3:** Nutrient values of child-targeted products for which label information was collected according to NOVA groups.

For 100 g/ ml Food	NOVA Groups				F	p
	NOVA 1 (Unprocessed or minimally processed food)	NOVA 2 (Processed culinary ingredients)	NOVA 3 (Processed food)	NOVA 4 (Ultra-processed food)		
	$\overset{―}{x}$ + SD	$\overset{―}{x}$ + SD	$\overset{―}{x}$ + SD	$\overset{―}{x}$ + SD		
**Energy (kcal)**	181.21 ± 165.34^34*^	286.00 ± 0.00	294.58 ± 133.19^1*^	359.52 ± 165.91^1*^	15.99	0.000*
**Fat (g)**	3.64 ± 6.64^34*^	0.00 ± 0.00	14.42 ± 7.22*	13.55 ± 12.82*	9.45	0.000*
**Saturated fat (g)**	1.14 ± 1.80^4*^	0.00 ± 0.00	5.00 ± 4.35	6.77 ± 7.00^1*^	10.19	0.000*
**Carbohydrate (g)**	28.00 ± 28.80^24*^	81.00 ± 0.00^13*^	21.50 ± 18.90^2*^	54.20 ± 27.20^1*^	18.73	0.000*
**Sugar (g)**	10.19 ± 16.29^24*^	76.00 ± 0.00^134*^	2.67 ± 2.31^24*^	36.96 ± 24.92^123*^	26.01	0.000*
**Fiber (g)**	4.67 ± 6.81^24*^	0.00 ± 0.00^13*^	4.67 ± 6.12^24*^	1.62 ± 2.29^13*^	20.20	0.000*
**Protein (g)**	7.38 ± 7.94^234*^	0.00 ± 0.00^134*^	16.00 ± 10.44^124*^	4.64 ± 3.54^123*^	37.40	0.000*
**Salt (g)**	0.11 ± 0.25^34*^	0.00 ± 0.00^3*^	0.95 ± 0.54^124*^	0.43 ± 0.60^13*^	7.76	0.000*
**Total (n)**	42 (%5.4)	3 (%0.4)	12 (%1.5)	724 (%92.7)	781	

*The upper characters in the rows indicate the groups with statistically significant differences (p < 0.05) as a result of the NOVA groups’ post-hoc test.

**One-Way Analysis of Variance (ANOVA), LSD Post-Hoc test

### Evaluation of compliance with nutrient profile models

As shown in Table 4, 93.2% of the products in the sample did not comply with the WHO NPM-2023 criteria. Additionally, a statistically significant difference (p < 0.001) was evident between permitted and non-permitted products according to WHO NPM-2023 in terms of the nutrient values (energy (kcal), fat (g), saturated fat (g), carbohydrate (g), sugar (g), fiber (g), protein (g), salt (g)). Similarly, 92% of the products in the sample did not comply with the TR NPM criteria. Analysis revealed a statistically significant difference (p < 0.001) between the permitted and not permitted groups according to the TR NPM in nutrients contents of product excluding fiber and protein. When evaluated according to the UK FSA/Ofcom NPM, 81.3% of the products were classified as less healthy. Moreover, statistically significant differences (p < 0.005) was observed between the mean nutrient values of the healthy and less healthy groups.

**Table 4:** Nutrient averages of child-targeted products for which label information was collected according to Nutrient Profile Model (WHO, TR and UK FSA/Ofcom NPM).

For 100 g/ ml Food	WHO NPM		p*	TR NPM		p*	UK FSA/Ofcom NPM		p*
	Permitted	Not Permitted		Permitted	Not Permitted		Healty	Less Healty	
	$\overset{―}{x}$ + SD	$\overset{―}{x}$ + SD		$\overset{―}{x}$ + SD	$\overset{―}{x}$ + SD		$\overset{―}{x}$ + SD	$\overset{―}{x}$ + SD	
**Energy (kcal)**	155.81 ± 141.49	365.70 ± 160.51	0.000*	137.61 ± 122.50	369.93 ± 158.11	0.000*	156.98 ± 152.76	392.71 ± 140.68	0.000*
**Fat (g)**	2.77 ± 5.69	13.83 ± 12.73	0.000*	2.15 ± 3.36	14.03 ± 12.76	0.000*	3.20 ± 5.61	15.22 ± 12.80	0.000*
**Saturated fat (g)**	1.25 ± 2.67	6.85 ± 6.97	0.000*	1.16 ± 2.14	6.93 ± 6.99	0.000*	1.03 ± 1.21	7.66 ± 7.08	0.000*
**Carbohydrate (g)**	23.79 ± 25.66	54.91 ± 26.71	0.000*	21.74 ± 24.05	55.48 ± 26.40	0.000*	26.44 ± 27.23	58.34 ± 24.63	0.000*
**Sugar (g)**	6.04 ± 2.89	37.57 ± 24.96	0.000*	6.61 ± 2.98	37.92 ± 24.93	0.000*	8.85 ± 8.41	41.19 ± 24.22	0.000*
**Fiber (g)**	2.75 ± 5.77	1.77 ± 2.58	0.018*	2.29 ± 5.42	1.80 ± 2.58	0.484	2.70 ± 5.12	1.63 ± 2.04	0.014*
**Protein (g)**	7.72 ± 8.57	4.78 ± 3.79	0.000*	6.61 ± 7.59	4.84 ± 3.91	0.073	5.21 ± 5.72	4.88 ± 3.97	0.515
**Salt (g)**	0.15 ± 0.61	0.45 ± 0.61	0.001*	0.14 ± 0.23	0.45 ± 0.61	0.000*	0.22 ± 0.39	0.47 ± 0.62	0.000*
**Total (n)**	53 (%6.8)	722 (%93.2)	775 (%100)	62 (%8.0)	713 (%92.0)	775 (%100)	146 (%18.7)	635 (%81.3)	781 (%100)

*p < 0.05 significant.

**Independent samples t-test.

The UK FSA/Ofcom NPM scores of the collected products (n = 781) demonstrated a very strong positive correlation with energy (r = 0.756, p = 0.000); good positive correlation with fat (r = 0.604, p = 0.000), saturated fat (r = 0.678, p = 0. 000) and sugar (r = 0.622, p = 0.000); moderately positively correlated with carbohydrate (r = 0.532, p = 0.000) and weakly positively correlated with salt (r = 0.211, p = 0.000), but weakly negatively correlated with fibre (r = −0.156, p = 0.000).

As shown in Table 5, 50.8% of the child-targeted products were categorized in Nutri-Score group E and 19.2% were in the group D. Statistically significant differences (p < 0.001) were found across Nutri-Score categories for all nutrient averages of children’s products, as confirmed by post hoc analyses presented in Table 5.

**Table 5:** Nutrient values of child-targeted products for which label information was collected according to Nutri-Score groups.

For 100 g/ ml Food	Nutri-Score Groups					F	p
	A	B	C	D	E		
	$\overset{―}{x}$ + SD	$\overset{―}{x}$ + SD	$\overset{―}{x}$ + SD	$\overset{―}{x}$ + SD	$\overset{―}{x}$ + D		
**Energy (kcal)**	258.83 ± 174.53^BDE^*	77.53 ± 82.63^ACDE^*	226.74 ± 166.99^BDE^*	358.91 ± 93.34A^BCE^*	460.16 ± 69.13^ABCD^*	416.00	0.000*
**Fat (g)**	5.21 ± 8.98^E^*	1.51 ± 2.86^CDE^*	7.66 ± 11.22^B^*	9.67 ± 12.30^EB^*	19.38 ± 11.39^ABD^*	83.42	0.000*
**Saturated fat (g)**	0.83 ± 2.26^DE^*	0.76 ± 0.73^CDE^*	2.39 ± 3.67^B^*	3.42 ± 4.49^ABE^*	10.51 ± 6.88^ABCD^*	118.05	0.000*
**Carbohydrate (g)**	38.46 ± 27.93^BDE^*	13.53 ± 16.01^ACDE^*	32.78 ± 26.34^BDE^*	61.66 ± 24.68^ABCE^*	65.95 ± 16.28^ABCD^*	195.33	0.000*
**Sugar (g)**	4.21 ± 6.16^CDE^*	8.20 ± 3.86^CDE^*	14.36 ± 10.88^ABDE^*	36.31 ± 23.33^ABCE^*	49.44 ± 21.41^ABCD^*	162.30	0.000*
**Fiber (g)**	8.96 ± 8.25^BCDE^*	0.40 ± 1.63^ACDE^*	2.71 ± 3.91^ABDE^*	1.65 ± 2.67^ABC^*	1.70 ± 1.37^ABC^*	59.56	0.000*
**Protein (g)**	12.67 ± 9.78^BCDE^*	2.74 ± 2.02^ACDE^*	5.21 ± 5.91^AB^*	6.09 ± 4.81^ABE^*	4.65 ± 2.87^ABD^*	35.18	0.000*
**Salt (g)**	0.21 ± 0.48^DE^*	0.09 ± 0.08^CDE^*	0.37 ± 0.56^BD^*	0.56 ± 0.79^AB^*	0.50 ± 0.57^ABC^*	15.35	0.000*
**Total (n) (%)**	24 (%3.1)	121 (%15.5)	89 (%11.4)	150 (% 19.2)	397 (% 50.8)	781 (%100.0)	

*The upper characters in the rows indicate the groups with statistically significant differences (p < 0.05) as a result of the Nutri-Score groups’ post-hoc test.

**One-Way Analysis of Variance (ANOVA), LSD Post-Hoc test.

The Pearson correlation coefficient was used to examine the relationship between Nutri-Score values and nutrient averages (n = 775). A strong positive correlation was identified between Nutri-Score values and energy (r = 0.801, p = 0.000), as well as a good positive correlation between fat (r = 0.605, p = 0.000), saturated fat (r = 0.670, p = 0.000) and sugar (r = 0.651, p = 0.000). A moderate positive correlation was observed between the Nutriscore scores and carbohydrates (r = 0.590, p = 0.000), while salt showed a weak positive correlation (r = 0.208, p = 0.000). Conversely, fibre demonstrated a very weak negative correlation with Nutri-Score values(r = 0.0108, p = 0.003).

### Nutrient profile model and food processed degree

A statistically significant difference was observed between the NOVA classification groups (p < 0.001) concerning the categorization of child-targeted products as permitted or not permitted according to nutrient profile models(WHO NPM, TR NPM, UK FSA/Ofcom NPM) as shown in Table 6.

**Table 6:** NOVA groups according to the nutrient profile of the collected child-targeted products.

WHO NPM	NOVA Groups										p
	NOVA 1		NOVA 2		NOVA 3		NOVA 4		Total		
	n	%	n	%	n	%	n	%	n	%	
**Permitted**	24	66.7	0	0.0	4	33.3	25	3.5	53	6.8	p = 0.000*
**Not Permitted**	12	33.3	3	100.0	8	66.7	699	96.5	722	93.2	
**Total**	36	4.7	3	0.4	12	1.6	724	93.4	775	100.0	
**TR NPM**	**NOVA Groups**										**P**
	**NOVA 1**		**NOVA 2**		**NOVA 3**		**NOVA 4**		**Total**		
	**n**	**%**	**n**	**%**	**n**	**%**	**n**	**%**	**n**	**%**	
**Permitted**	23	63.9	0	0.0	4	33.3	35	4.8	62	8.0	p = 0.000*
**Not Permitted**	13	36.1	3	100.0	8	66.7	689	95.2	713	92.0	
**Total**	36	4.7	3	0.4	12	1.6	724	93.4	775	100.0	
**UK FSA/Ofcom NPM**	**NOVA Groups**										**p**
	**NOVA 1**		**NOVA 2**		**NOVA 3**		**NOVA 4**		**Total**		
	**n**	**%**	**n**	**%**	**n**	**%**	**n**	**%**	**n**	**%**	
**Healthy**	34	81.0	0	0.0	8	66.7	10	14.4	146	18.7	p = 0.000*
**Less Healthy**	8	19.0	3	100.0	4	33.3	620	85.6	635	81.3	
**Total**	42	5.4	3	0.4	12	1.5	724	92.7	781	100.0	

*Fisher Exact test.

A statistical difference was observed between the NOVA groups and the UK FSA/Ofcom NPM and Nutriscore nutrient profiling models of the child-targeted products (p < 0.000). The difference between the groups was subsequently analysed by post hoc tests and the results are presented in Table 7.

**Table 7:** UK FSA/Ofcom NPM scores and Nutri-Score scores according to the NOVA groups of the collected child-targeted products.

UK FSA/Ofcom NPM Score				
NOVA Groups	n	$\overset{―}{x}$ + SD	F	p
NOVA 1^234^*	42	−1.71 ± 5.25	53.814	p = 0.000
NOVA 2^13^*	3	13.00 ± 0.00		
NOVA 3^124^*	12	3.25 ± 6.88		
NOVA 4^13^*	724	12.63 ± 7.61		
**Total**	781	11.72 ± 8.21		
**Nutri-Score Score**				
**NOVA Grupları**	**n**	$\overset{―}{x}$+ **SD**	**F**	**p**
NOVA 1^24^*	36	−0.31 ± 6.90	46.138	p = 0.000
NOVA 2^3^*	3	18.00 ± 0.00		
NOVA 3^24^*	12	4.25 ± 5.33		
NOVA 4^13^*	724	17.40 ± 9.69		
**Total**	775	16.37 ± 10.32		

*The upper numbers in the rows indicate the groups with statistically significant differences (p < 0.05) as a result of the NOVA groups’ post-hoc test.

**One-Way Analysis of Variance (ANOVA), LSD Post-Hoc test.

## Discussion

This study is, to our knowledge, the first time in Türkiye to assess the nutritional quality of packaged foods and beverages marketed for children aged 3 years and older using 4 different nutrient profile models (TR NPM, WHO NPM-2023, UK FSA/Ofcom NPM and Nutri-Score labeling system) and evaluate their processing degree using the NOVA classification. Among the packaged foods and beverages specifically marketed to children and included in our sample on the basis of predefined child-targeting criteria, the great majority were ultra-processed and of sub-optimal nutritional quality. When assessed with the WHO and other nutrient-profiling models, most of these child-targeted products would be considered unsuitable for marketing to and regular consumption by children.

Marketing and nutrition labelling are two of the most influential factors shaping consumers’ perception of a food’s healthfulness and their purchase decisions [40]. Multitude persuasive marketing techniques influence children’s dietary habits, and consumption pattens. Existing evidence shows that exposure to marketing of nutritionally poor products significantly increases children’s preference for energy-dense, nutrient-poor foods and beverages [40]. The most common marketing techniques identified in this study were the use of childish images (47%), playful names and fonts (34.5%), and child-friendly product shapes (28%). Previous studies have similarly noted the frequent use of child-targeted naming, characters, cartoon mascots, and appealing product shapes as primary marketing tactics [41–43]. These findings highlight the food industry’s strategic use of intensive marketing methods designed to capture children’s attention and enhance product appeal. “Pester power” is a term used to describe “children’s influence over adult purchasing”. Children are influenced by marketing techniques in foods, even if they cannot buy them themselves and force their parents to buy products that they would not normally buy [44].

Our results indicate a prevalence of child-targeted marketing strategies primarily in categories such as chocolate, sugar, energy bars, sweets (38%), and cakes, cookies, and sweet bakery products (23%). This finding is consistent with the conclusions of previous studies in the literature [19,41,42]. As previously observed by other researchers, the products targeted at children in our study were predominantly sugar-based products.

The high levels of sugar, saturated fat, and total fat observed in these child-targeted products raise significant concerns. Our findings demonstrate that ultra-processed foods have significantly higher energy, fat, saturated fat, sugar, and salt content, and significantly lower protein and fiber content compared to minimally processed foods (p < 0.001), as indicated in Table 3. These findings are consisted with prior studies indicating that ultra-processed foods contain a higher percentage of estimated calories from carbohydrates and added sugars compared to non-ultra-processed food [9,45]. Additionally, these foods are typically lower in fiber and protein, and higher in sodium, iron, vitamin E, and folic acid [9].

An extensive research has demonstrated that the consumption of processed foods among children is reaching alarming levels, with a considerable proportion of their caloric intake derived from ultra-processed foods [9,10]. Reducing the consumption of ultra-processed foods has been shown to significantly decrease obesity risk in children and adolescents. Furthermore, the consumption of sugary products, salty snacks and chips, which are specifically targeted at children and are a popular choice among this demographic, should be discouraged during the preschool years due to the high saturated fat, salt and sugar content, which increases the risk of obesity, cardiovascular disease and other non-communicable chronic diseases [46].

Despite clear recommendations by the WHO Regional Office for Europe against promoting unhealthy products through any marketing strategy [20], our findings are consistent with other studies in the literature [41,43,47], our study found that 93.2% of child-targeted products in Türkiye failed to meet WHO NPM-2023 criteria and 92% were non-permitted category according to TR NPM criteria. Similarly, according to the UK FSA/Ofcom NPM, 81.3% were found unpermitted category for marketing. Our main finding in this study was that the majority of child-targeted products available on the Turkish market do not comply with the WHO recommendations. However, they can continue to be marketed in grocery stores. This highlights the urgent need for stricter regulations to limit marketing unhealthy foods to children and incorporate packaging strategies into legislative policies.

The majority of the analyzed child-targeted products were classified within Nutri-Score groups D and E (70%), with 92.7% identified as ultra-processed. If processed and ultra-processed foods are considered less healthy according to the NOVA classification system, the rate can be expressed as 94.2%. Comparable results from a Brazilian study [48] indicated NOVA’s classification as more stringent than NPM, categorizing a higher proportion of child-targeted products as less healthy. Our findings align closely with these results, underscoring the necessity for targeted public health interventions.

Additionally, there was a significant association between foods classified as prohibited or less healthy according to the nutrient profile models (NPMs) and ultra-processed food categories (p < 0.001, Table 6). Another significant finding of our study was ultra-processed products also had higher Nutri-Score values, indicating a less healthy nutritional profile (p < 0.001) (Table 7). Previous research has shown that poor Nutri-Score ratings correlate with reduced diet quality [49].

The findings of our study also demonstrate a high degree of consistency between the nutrient profile models used, and the Nutri-Score and the NOVA classification. However, it should be noted that 14.4% of the foods classified as healthy when evaluated with the UK FSA/Ofcom NPM in our sample were in the NOVA 4 category. Although food processing and food quality are not the same thing, they are complementary elements of a healthy diet. The findings of our study indicate that the UK FSA/Ofcom NPM may be insufficient to identify all unhealthy foods. This may be because UK FSA/Ofcom NPM consulted the food industry more during its process [20].

Similar results were shown in comparable studies conducted in other countries [41,43,47]. A recent Swiss study analyzing 735 child-targeted products found that 92.8% did not meet WHO NPM-2023 criteria, with 58% scoring D or E on the Nutri-Score and 91.8% categorized as ultra- processed [41]. It was indicated that products with a lower degree of processing and products that met the WHO NPM-2023 criteria exhibited a superior Nutri-Score value [41]. Similarly, Richonnet et al. [43] analyzed 1155 products in France, reporting that 94.88% failed to meet WHO NPM criteria, with most products scoring D or E on Nutri-Score (58.7%) and 87.7% being ultra-processed. The majority of cheeses, cakes, salty snacks, and confectionery were found to have a Nutri-Score of D, while the majority of soft drinks, biscuits, and chocolates were found to have a Nutriscore of E [43].

A Slovenian study evaluating 438 child-targeted products according to WHO NPM-2015 similarly identified that 93% of products were unsuitable for marketing to children [47]. In Belgium, Aerts and Simits [42] assessed 372 child-targeted products using the UK FSA/Ofcom NPM, finding 89.2% to be non-permissible for marketing. These international findings reinforce the results from our Turkish market study and underscore the necessity of stronger regulations limiting child-targeted marketing of unhealthy foods.

## Conclusions

Our study indicates that most child-targeted packaged foods and beverages marketed to children in Türkiye are ultra-processed and had poor nutritional quality. This finding highlights the urgent need to implement stricter marketing restrictions and labeling regulations to protect children from unhealthy dietary choices. Furthermore, implementing policies that clearly communicate nutritional quality and processing levels through front-of-pack labeling, such as Nutri-Score and NOVA classification could motivate parents to make more informed decisions and encourage manufacturers to develop healthier product formulations. It may also be beneficial to provide information regarding the degree of processing on product packaging. Changing aspects of the food environment that facilitate the consumption of UPF products, such as availability, marketing, and price, and requiring the use of warning labels on products if the content of nutrient of concern exceeds specified levels, may also be effective strategies.

Public health measures must be implemented to restrict the marketing of unhealthy products to children. Further research is recommended on how and which public health policies can effectively protect children from the negative effects of marketing. Also, further research needed in evaluation of children’s actual dietary habits and product consumption patterns.

It should be noted that our study has some limitations. The lack of fiber information on some product labels may have resulted in minor discrepancies in the Nutri-Score and UK FSA/Ofcom NPM calculations, as the proportions of fruits, vegetables, and nuts were estimated from the ingredients section. Additionally, the accuracy of the nutrient and ingredient lists on product labels was assumed based on the study’s design. Furthermore, our data and analyses do not include the dietary habits and consumption patterns of these products by children in Türkiye.

Notwithstanding the limitations above, the study has several notable strengths. To the best of our knowledge, this is first study in Türkiye to comprehensively evaluate the nutritional quality and degree of processing of packaged products marketed specifically to children aged 3 years and older. The extensive data collection, involving multiple visits to numerous grocery stores and chain market branches throughout Ankara, ensures comprehensive coverage and representation of products marketed to children and reflecting the diversity of the Turkish market. Moreover, using internationally validated tools that take into account various dimensions of nutritional quality (Nutri-Score, NOVA, WHO NPM, and UK FSA/Ofcom NPM) enhances the reliability of our results and facilitates direct comparison with international studies.
